# Case managers for older persons with multi-morbidity and their everyday work – a focused ethnography

**DOI:** 10.1186/1472-6963-13-496

**Published:** 2013-11-27

**Authors:** Markus Gustafsson, Jimmie Kristensson, Göran Holst, Ania Willman, Doris Bohman

**Affiliations:** 1 School of Health Science, Blekinge Institute of Technology, Karlskrona SE-379 71, Sweden; 2 Department of Health Sciences, Lund University, Lund SE-221 00, Sweden; 3 Department of Care Science, Malmö University, Malmö, Sweden

**Keywords:** Case manager, Case management, Continuity of care, Ethnography, Focused ethnography, Intervention, Multi-morbidity, Older persons, Thematic analysis

## Abstract

**Background:**

Modern-day health systems are complex, making it difficult to assure continuity of care for older persons with multi-morbidity. One way of intervening in a health system that is leading to fragmented care is by utilising Case Management (CM). CM aims to improve co-ordination of healthcare and social services. To better understand and advance the development of CM, there is a need for additional research that provides rich descriptions of CM in practice. This knowledge is important as there could be unknown mechanisms, contextual or interpersonal, that contribute to the success or failure of a CM intervention. Furthermore, the CM intervention in this study is conducted in the context of the Swedish health system, which prior to this intervention was unfamiliar with this kind of coordinative service. The aim of this study was to explore the everyday work undertaken by case managers within a CM intervention, with a focus on their experiences.

**Methods:**

The study design was qualitative and inductive, utilising a focused ethnographic approach. Data collection consisted of participant observations with field notes as well as a group interview and individual interviews with nine case managers, conducted in 2012/2013. The interviews were recorded, transcribed verbatim and subjected to thematic analysis.

**Results:**

An overarching theme emerged from the data: Challenging current professional identity, with three sub-themes. The sub-themes were 1) Adjusting to familiar work in an unfamiliar role; 2) Striving to improve the health system through a new role; 3) Trust is vital to advocacy.

**Conclusions:**

Findings from this study shed some light on the complexity of CM for older persons with multi-morbidity, as seen from the perspective of case managers. The findings illustrate how their everyday work as case managers represents a challenge to their current professional identity. These findings could help to understand and promote the development of CM models aimed at a population of older persons with complex health needs.

## Background

There is interest in how Case Management (CM) should be designed in order to address the complex needs of older persons with multi-morbidity. To promote the development of CM models, there is a need for more research to provide detailed descriptions of how this form of everyday management takes place. This research is needed in order to gain a better understanding of its underlying mechanisms [1]. CM as a model aims to improve the co-ordination of different services, such as health and social care [2]. CM has become a way of intervening in a fragmented health system that is moving towards non-optimal care for older persons [3]. Several studies [1,3-5] have investigated the effects of CM interventions for older persons, focusing mainly on healthcare costs and healthcare use. However, the results have been inconsistent and range from positive outcomes to no effect whatsoever [1,3-5]. Moreover, the studies are often described in less detail, making it difficult to comprehend what has actually taken place as an intervention [1,3,4]. Hence, there is a need for additional knowledge about possible mechanisms that could affect a successful CM intervention [1]. One way to advance this knowledge could be to investigate the practice and experiences of those who work according to the model, i.e. case managers, cf. [3,6]. This knowledge is important since interpersonal and contextual factors, as well as unknown factors, could contribute to the success or failure of a CM intervention [3]. Deeper knowledge of everyday practice among case managers could lead to better understanding. This knowledge could also help to advance the development of CM models designed for an older population with complex health needs.

The proportion of older persons in Europe is increasing, highlighting the importance of a capable health system that can cope with complex needs [7]. Within this aging population, a substantial number of older persons have multiple, independent diseases, i.e. they can be described as having multi-morbidity [8,9]. The population of older persons with multi-morbidity is expected to grow in line with increased longevity and improved living conditions [10]. These older persons could experience difficulty coordinating care efforts and they often lack a complete overview of their own health and social care contacts. Difficulty adhering to a complex health system could lead to fragmented care, i.e. a lack of continuity [11,12]. Continuity of care for these older persons usually includes all their points of contact with the health system in addition to their direct contact with the care providers [13]. The lack of co-ordination and the lack of a holistic approach within health and social care for older persons result in unsatisfactory care [14]. Consequently, this unsatisfactory care coordination can increase the older person's dependence on others, such as family members [11]. Family members often assume a great deal of responsibility for the older person's well-being [15]. To reduce this fragmentation of care in the current health and social care system, it has become increasingly important to develop and evaluate models to improve continuity of care [16]. A management model that addresses this issue is CM, where case managers guide and assist people in their contact with health and social care representatives [2].

**Figure 1:**
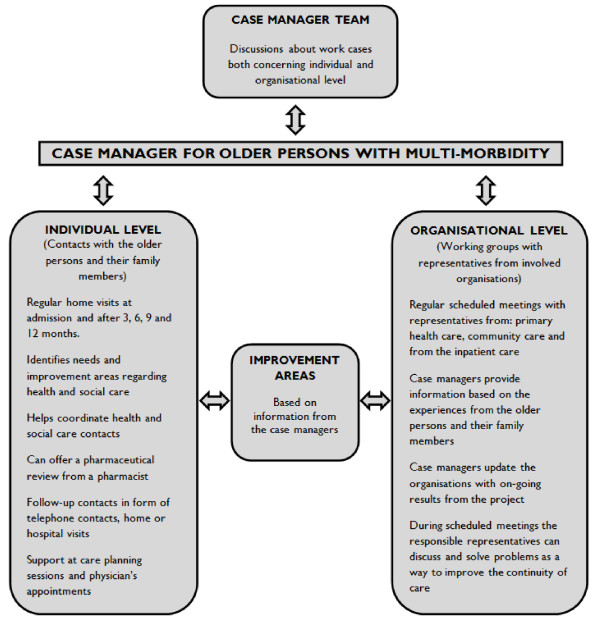
Design of the Blekinge case management intervention.

Case management is a generic name for models that are used to co-ordinate care for people with complex needs, such as older persons with multi-morbidity [2]. The Case Management Society of America has defined CM as "… *a collaborative process of assessment, planning, facilitation, care coordination, evaluation, and advocacy for options and services to meet an individual's and family's comprehensive health needs through communication and available resources to promote quality, cost-effective outcomes.*" [17]. There are different CM models that address aspects ranging from purely financial matters to a more holistic approach to the individual's needs [2,5,18-20]. These models may also differ in terms of how active the clients are supposed to be, the extent to which management services are handled within the CM team, and how intensively the case managers work with their management activities [2,5,18-20]. Earlier research has explored the whole or parts of different CM models, viewed from the perspective of the case managers [21-25]. However, the CM model in this study, described further in the next section, differed in design. This difference was due to the case managers' potential to directly influence parts of the organizational structure within the health system (See Figure 1). This organizational influence was performed during regular meetings of working groups made up of representatives from the organizations involved. The purpose of these working groups was to improve continuity of care for older persons with multi-morbidity.

In 2011–2013, an ongoing CM intervention took place in the county of Blekinge in southern Sweden. The purpose was to improve continuity of care for older persons with multi-morbidity. The CM intervention consisted of activities performed on two levels (See Figure 1). One level was the organizational level, where case managers identified areas in which continuity of care could be improved and they presented these areas to representatives from the organizations involved. The second level was the individual level, where case managers identified the differing and changing needs of older persons and coordinated these needs in such a way that other health and social care contacts would perform the tasks required. One of the case managers' main functions therefore was to improve continuity of care for older persons with multi-morbidity. Each case manager was assigned to one of the five municipalities in the county of Blekinge. The CM intervention did not belong to any current healthcare organization and took place within a completely new and temporary organization.

Earlier research [1,3,16,26] highlights the importance of CM interventions that aim to improve continuity of care for older persons. This research also acknowledges that there are inconsistencies in results regarding whether CM interventions are effective or not for this purpose. Many of these CM interventions are also described in less detail, making it difficult to comprehend what has actually taken place as an intervention [1,3,4]. To further the development of CM models, there is a need for additional research that explores how CM is being carried out in practice by the case managers. This is important since we need more knowledge about mechanisms, both contextual and interpersonal, that may affect the success or failure of a CM intervention [3]. Furthermore, the CM intervention in this study is conducted in the context of the Swedish health system. Prior to this intervention, this was a context that was unaccustomed to this kind of coordinative service. The aim of this study was to explore the everyday work undertaken by case managers within a CM intervention, with a focus on their experiences.

## Methods

### Design

The study design was inductive and qualitative, utilizing an ethnographic approach. Data collection consisted of participant observations with field notes, a group interview and individual interviews with nine case managers, all conducted in 2012/2013. The ethnographic approach is particularly suited when focusing on a group of persons who all have something in common [27]. This applies to the participants in their everyday work as case managers. Ethnography as a research method is characterized by the researcher's role as an instrument, with a focus on observation and data collection that includes different sources, i.e. data triangulation. This data collection is conducted in the participants' normal environment [28]. The ethnographic perspective allows the researcher to become part of the specific context within which case managers operate and to learn from these persons during the course of their everyday work [28]. Throughout this study, the principles of focused ethnography [29] were applied. This is an interpretive approach that allows an in-depth, exploratory study of experiences from both an emic perspective, i.e. the inside view of the case managers, and an etic perspective, i.e. the outside view of the researcher [30]. This approach is characterized by episodic participation observations and is well suited to health research as it allows for predetermined research questions [29].

### Participants and study setting

Participants in this study were selected by means of purposeful sampling [31]. The prerequisite for participation in this study was work experience as a case manager within the Blekinge CM intervention. At the start of the intervention in April 2011, ten case managers were involved. Three of these ten case managers had terminated their involvement prior to this study. At the start of the study, seven case managers were still active in the intervention and in September 2012 they were recruited for a group interview, individual interviews and participant observations (See Table 1). Two of the three inactive case managers were also included for the individual interviews. One of the three inactive case managers did not respond to the invitation. A total of nine participants were included and their age ranged from 40 to 61 years with a mean age of 50 years. They had different professional backgrounds: three were nurse managers, two were registered nurses, three were assistant nurses and one was an occupational therapist. Their professional experience in their current professions ranged from 4–34 years, with a mean length of experience of 18 years. None had previous experience as a case manager before entering the intervention.

**Table 1:** Overview of interviews and observations

	**Individual interviews**	**Group interview**	**Observations**
*Number of participants* (n)	9	7	
Active case managers	7	7	7
Inactive case managers	2		
*Interview duration* (minutes)			
Active case managers	47-111	103	
Inactive case managers	25-41		
*Number of observations* (n)			36
Time range (hours)			0.75-8
Total time (hours)			125

The study was performed in Blekinge, a county in southern Sweden with a population of around 150,000 and comprising of both rural and urban areas. Blekinge has one regional hospital and one local hospital. The health system consists of primary healthcare, community care and inpatient care. Although the county councils are the regional healthcare providers, the five municipalities are responsible for the care of older persons living in nursing homes or in their own homes [32]. In the Swedish health system, there are to our knowledge no CM services incorporated aimed at assisting older persons with multi-morbidity with continuity of care.

### Procedure

The first author (MG) began collecting data through participant observations conducted between June 2012 and January 2013. There are various participant observation approaches and in this study the emphasis was on the 'observer as a participant' approach. This means the researcher conducted observations during predetermined periods and did not become fully immersed in the lives of those being observed [33]. These observations were performed as part of the case managers' everyday work, during their weekly follow-up meetings and during reflective meetings. These reflective meetings dealt with the participants' experiences of being a case manager and the first author acted as both observer and moderator.

In line with the ethnographic approach [28-30], the first author observed the daily work of the case managers. Observations were performed while trying to keep an open mind and reflecting on the situations that emerged. There were 36 observation periods involving all active case managers (See Table 1). The duration of the observations depended on the participants' availability and the nature of the activities that were being observed. During the later observations, the first author became familiar with the daily situations encountered by the case managers and decided not to perform any more observations. During these observations, field notes were taken continuously and included information about the date, place and time of the observation, the environment, the participants, verbatim verbal exchanges, personal reflections and a presentation in chronological order of what happened during the observation. All this information was collected to provide a detailed description of the observed situation [33].

Before the interviews began, a pilot group interview was conducted in September 2012 with five registered nurses. As they all had experience of working with older persons with multi-morbidity they were found to be of value for a pilot interview. During this pilot interview, the interview questions were tested for feasibility and the ability to initiate in-depth accounts. Minor revisions were made. A group interview was later performed in September 2012 with all seven currently active case managers (See Table 1). The purpose of the group interview was to explore the case managers' individual experiences but in a group context. Following the group interview, the in-depth individual interviews were conducted in September and October 2012 with nine case managers (See Table 1). The group interview was conducted by two of the authors (MG and DB) at their workplace, i.e. the university, where DB acted as moderator. The individual interviews were performed by the first author at the case managers' workplace (n = 7) and at the university (n = 2). A thematic interview guide [34] was used to guide both the group and individual interviews and supported three themes: (1) The case managers' experiences of their everyday work; (2) The case managers' encounters with other professions within the health system; (3) The case managers' experiences of continuity of care for older persons within the health system. These themes were based on the aim of the study and were chosen in the light of the circumstance that this CM intervention was being tested in the Swedish health system. Prior to this study, the health system was unaccustomed to case management services for older persons with multi-morbidity.

### Data analysis

In line with the ethnographic approach [28-30], the informal analysis commenced during the observations. Field notes from the observations were critically reflected on by the authors (MG, JK, GH and DB) as part of a continuous, iterative process. This was done in order to gain a deeper understanding of the case managers' everyday work. The continuous analysis took place in the light of the aims of the study. It influenced the observations and led to the observer (MG) initiating informal interviews with the case managers. These informal interviews were initiated since new insights needed to be explored to a greater extent in the field. The field notes were also used to form a narrative of a typical working day of a case manager. The aim of this narrative was to illustrate a case manager's everyday work in order to facilitate the reader with a deeper understanding of the context. The field notes also contributed to a pre-understanding, which was present during the formal analysis.

The formal analysis, i.e. the analysis of the interview material, first began when the observations were completed. Data analysis of the interview material was influenced by the description of thematic analysis by Morse and Field [35]. All interviews were transcribed verbatim. This marked the first step in the formal analysis as it was the first author who transcribed part of the interviews. According to Klein [36], transcription is not only a mechanical act but also an analytical act, where the researcher's pre-understanding can affect the transcription process. The remaining interviews were transcribed by a secretary skilled in transcribing research material. All the interviews were listened to and read through several times by the first author to gain an overall understanding of the content. The other authors (JK, GH and DB) read a sample of the interviews to acquire an understanding of the data. The overall impressions of the participants' experiences were noted and discussed by the authors (MG, JK, GH and DB). The first author regularly took a step back to reflect on the interviews as a whole. During this continuous iterative process three different themes later emerged, linking substantial portions of the interviews. These themes began to reoccur across the texts and no new themes emerged. The similarities and differences in these emerging themes where discussed by the authors and a consensus was reached (MG, JK, GH and DB). An overarching theme was lifted from the themes and was subsequently acknowledged by all the authors. Due to the extensive data collection (See Table 1) and as no new themes emerged from the interview data reflecting the aim of the study, the authors decided to finalise the analysis of the data.

### Ethical considerations

This study was conducted in compliance with the established ethical guidelines of the Declaration of Helsinki [37]. All participants received written and verbal information about the study and its purpose as well as notification that the data would be treated in confidence. All participants were informed that they could discontinue their participation in the study at any time without being required to give a reason. All recorded material was transcribed and coded to ensure data anonymity. Data is stored securely and anonymously in compliance with the Data Protection Act (SFS 1998:204). Ethical approval was applied for and granted by the Regional Ethical Review Board in Lund (Ref. No. 2012/228).

## Results

The results contain findings that focus on how case managers experience their everyday work within a CM intervention. This section presents findings from the observational data, i.e. participant observations and field notes, as well as the thematic analysis of the interview data.

### Observational data

#### A case manager's typical working day

A typical working day for a case manager involves coming to the office in the morning. At the office she starts to plan the day by looking in the diary to see what has been booked. By searching for information in the documentation system and on the Internet she finds information that could be of help in the upcoming assignments. She calls different health professionals to investigate the assignments. She then contacts another case manager in the team who has professional experience of the context in which a particular assignment has occurred. She then consults and receives advice on the assignment in question.

After planning has been completed, she calls one of the older persons whom she plans to visit during the morning to make sure he is at home and can receive a home visit. She drives to the older person's house, which is situated in a more rural area. During the home visit she meets the older person and his wife. She listens to what the older person has to say and tries to assess the perceived needs of the older person by asking probing questions. She informs the older person and his wife about their rights and what kind of help they could expect from the health system. She then guides him to the appropriate health care contact. She helps the older person to fill in a questionnaire about his current health situation, which will later be used for evaluation purposes. At the end of the home visit they agree to contact each other in two weeks to see if he has received the help he needs.

After the home visit, she drives back to town to attend a meeting with the municipal working group. The working group is overseen by a project management team, which is in turn overseen by a steering committee made up of senior representatives from the organizations involved. The reason for this arrangement is to manage the project, collect information from the case managers and make decisions about possible improvements within the organizations that are represented. She presents the areas that require improvement to the representatives in the working group. By way of illustration, she takes real examples from her everyday practice and emphasizes the importance of the older person's individual needs. During the working group meetings she hears about the organizational changes that are taking place within the municipalities. She writes these down in a notebook in order to provide the older persons with information. After the meeting with the working group she returns to the office and begins to document the day's activities in the documentation system. Her phone rings. It is one of the older persons who needs advice on how to obtain help. She listens to what the older person has to say and then guides him to the appropriate healthcare contact. Before ending her working day she calls one of the older persons she helped the previous week to see if the person has received the help he required and to find out if it had been satisfactory.

### Thematic analysis

**Figure 2:**
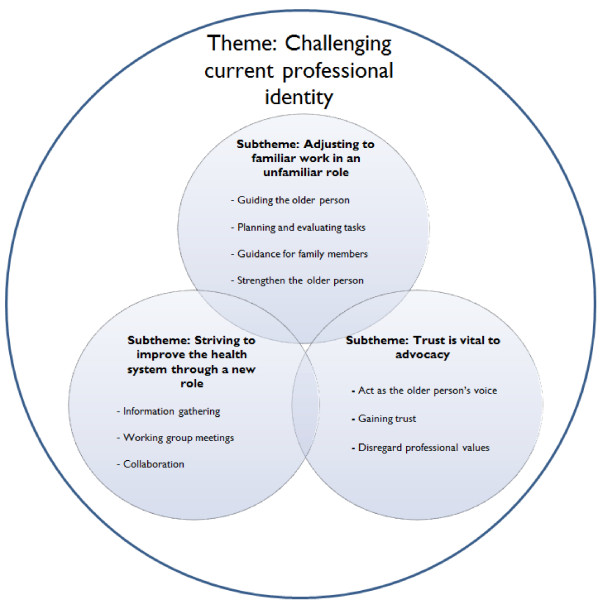
Theme and sub-themes.

From the interview data, an overarching theme emerged: Challenging current professional identity, along with three sub-themes: 1) Adjusting to familiar work in an unfamiliar role; 2) Striving to improve the health system through a new role; 3) Trust is vital to advocacy (See Figure 2).

#### Challenging current professional identity

The overarching theme of challenging current professional identity was embraced by the participants as they adjusted to familiar work but in an unfamiliar role as case managers. When performing their tasks they utilised their pre-understanding as well as the knowledge residing within the case manager team. As they performed this familiar work they were subjected to different conditions that govern how they should or should not perform the tasks. Adherence to these diverse conditions, both as an individual and as a team member, challenged their current professional identity. The theme also involved the participants continuous strive towards improvement of the health system. They had all been assigned a new role that offered them a unique potential to improve but also presented them with new challenges. While striving to bring about improvement within the organizations they needed to adapt to ways that would enable them to do so. Trying to adapt to these new conditions challenged their current professional identity. The overarching theme was also reflected in the need among the participants to trust the older person in order to act as their advocate. They were required to set aside their own professional values and experiences of the health system in order to advocate from the older person's perspective. The new role as case manager was shaped as time passed and their experience developed. They all had to challenge their professional identity when trying to come together as a case manager team and work towards common goals.

"Yes, I think the role of case manager changes all the time. There has never been any clear description of what to do or not to do. It constantly takes a different path and we try to forge ahead. The better you become, the more you know where to set limits and what your remit is. In the team you listen to what they're doing and how they work to avoid everyone going off in different directions. But now I think we're trying to gather ourselves. We might need to stop in order to avoid everyone going in different directions and come together instead. I think the role of case manager changes over time. The longer you are a case manager, the more you become a case manager and not something else." (Case manager 2)

##### Adjusting to familiar work in an unfamiliar role

The participants all stated that their everyday work as a case manager was not unfamiliar in relation to previous tasks in their professions. However, they all felt that they now performed this familiar work in the completely new and unfamiliar role as case manager. This new role gave rise to other conditions that governed how they should or should not perform this familiar work. This work consisted of tasks they were expected to perform as a part of their everyday work. Performing these tasks in an unfamiliar role made them challenge their current professional identity.

One of these familiar tasks was to guide the older person through the health system. This was done by providing information about available services and directing the older person to the appropriate healthcare contact. They described how in the past they had met older persons with multi-morbidity within their professions and had acquired experience of how to guide them. They knew what other health professionals were expected to do. They interacted with numerous professions and the needs of the older person would guide them to the right profession. They often advised the older persons to initiate the contact directly but sometimes they handled these contacts due to the older person's limited capacity.

When providing guidance they made use of the extensive knowledge within the case manager team. They all came from different backgrounds and had different organizational and professional affiliations. This was considered helpful as they could use the range of professional experience within the team when they needed more knowledge to guide the older persons. This was described as integration of the team's collective expertise. They felt strong knowing that they had a broad expertise base behind them in their case manager team. As they integrated the team's expertise they also needed to put this knowledge into their own professional perspective. They reported that knowledge from the other team members became intertwined with their own pre-understanding, which contributed to their personal development.

"My team members have been adding to my knowledge, I have received so much input from the different professions and this has given me strength. Now I can present suggestions and ideas that I might not have been able to do at the beginning." (Group interview)

When presenting themselves as case managers to the older persons they often had to clarify the role of case manager. The reason was that the older persons misunderstood how they could receive assistance. The case managers needed to explain that they were not taking the place of other professions; they mainly coordinated health and social care contacts. They tried to strengthen the older persons by encouraging them to contact the health and social care services personally. They described this as 'standing beside the older persons and supporting them in their own actions'. They felt it was their duty to always direct an older person to another health or social care contact if possible as they did not want the older person to be solely dependent on them to perform the various services. Because the time span of the CM intervention was limited, they did not know whether or not their services would be implemented. This caused a feeling of uncertainty, particularly in those cases where they did not know the future outcomes of the processes that their own actions had initiated.

They sometimes felt powerless in the unfamiliar role of case manager. They were outside the health system and could not always work directly to alleviate the older person's problem. It was not their responsibility but the responsibility of other health professions. Standing outside the organization and not belonging to any profession sometimes made it unclear what they were expected to do. They felt frustrated about not having clear directives regarding what a case manager should or should not do in certain situations. In their current professions they were all accustomed to the possibility of performing more direct tasks to alleviate certain problems for their clients. As case managers they had to regularly take a step back, which meant not performing certain direct tasks, which challenged their current professional identity.

"I don't even have a sticking plaster with me to put on the patient." (Case manager 8)

The case managers were each assigned to a specific municipality and this made it easier for them as they felt they had more extensive knowledge about health and social services in that particular municipality. They had also developed contacts with different professions in the municipality, which made it easier as they knew where to seek the help required. When trying to meet the needs of older persons, the case managers cooperated with different professions within the health system and with voluntary organizations. Working in the health system, they met a wide range of older persons with multi-morbidity. Some were bedridden and others were described as being full of energy and too busy living their lives to even speak to the case managers. They also reported widespread loneliness among older persons. When they encountered loneliness they attempted to determine if the loneliness was a problem for the older person. If so, they often tried to direct the older person to voluntary support organizations that offered social companionship. Although they were saddened by the fact that so many of the older persons were lonely, they did not regard it as part of their remit as case manager to offer social support because of loneliness.

"You cannot visit people every day just because they are alone. Then there's something lacking in the system. This loneliness problem is difficult but it's too big to solve as a case manager." (Case manager 9)

As case managers, they were required to enlist older persons for the CM intervention and they adopted different strategies to achieve this. One of these strategies was to select older persons by using a register, after which requests by letter were sent out with information about the project. They then contacted the older persons by telephone to ask if they were interested in the services of a case manager. This approach did not feel natural in the sense that the older persons had not sought the assistance of a case manager personally. This also meant that a number of the persons they contacted did not require their services. This recruitment process was unfamiliar to them as in their current professions they were not used to selling themselves. Instead, the clients came to them with the clear aim of seeking their assistance. However, they also felt that some of the older persons they had enlisted from the register might never have contacted them otherwise and they saw this aspect of the process as important when trying to identify those in need of their services. They felt that those in greatest need of a case manager often came to their notice after they were alerted by other health professionals or when they were contacted directly by the older persons. They described how they occasionally tried to provide their services to older persons who might not have needed those services, making them feel like a salesperson.

"You need to explain and try to sell, almost like a vacuum cleaner salesman, almost religiously, when you come along with your big bag and start pulling out brochures!" (Case manager 6)

Another familiar task expressed by the case managers was planning and evaluating actions based on the older person's needs. They felt they had considerable freedom to plan their working day, something which they were not accustomed to in their current professions. Sometimes they had to investigate the rights of the older person and examine decisions that had been made in the past by the different organizations. They regularly examined whether the older person had received the services that had been decided and if these services had been performed satisfactorily. The frequency of these evaluations was decided by the older person or in conjunction with the case manager when they saw a need to monitor the older person. They emphasized prompt service as the older persons were considered frail and their health could deteriorate rapidly.

"…then I felt yesterday, because it had been several weeks since I last called, that I should call and ask how it went. Well, by then he had died." (Case manager 5)

When planning their tasks they listened to what the older persons told them and tried to identify their needs during the conversation. They felt they had time to sit down and really listen to what the older person had to say. This was something they were not used to in their current professions, as normally they did not have the time to sit down and just listen. When they performed a needs assessment they often had a similar way of asking questions regarding the older person's current health status and their daily activities. They also sought to adopt a holistic perspective when assessing the needs of the older persons and they defined this perspective as having the time to listen to everything the older person had to say. This challenged their current professional identity as they were used to focusing on the aims that formed part of their professional responsibilities. They emphasized the importance of seeing the older person from a different point of view and acknowledged that they were all influenced by their pre-understanding. This pre-understanding sometimes caused them to search for needs based on their professions and not from the point of view of the older person. They all emphasized how important it was that the needs originated from the older person and were not clouded by their pre-understanding.

"I also think there's some excitement in it. You don't have a solid … moulded shape. You shape it according to the needs that emerge." (Case manager 2)

As case managers, they were a source of guidance for the older person's family members. In most cases the family member was the spouse but it could also be a daughter or a son. They supported the family members in their thoughts and in their struggle to deal with health and social services. The regularity of contact with the family members varied, ranging from no contact whatsoever to the point where the family member was the main driving force. The degree of contact depended mostly on the older person's health. Sometimes they were too sick to remain in contact and the family members assumed a more active role. In some cases the case managers stated that it was the family members who had persuaded the older person to accept their services. They believed that to a certain extent they relieved family members of the burden of responsibility. They also stated that the family members felt more secure knowing that someone was safeguarding the interests of the older person. In some cases the family members did not want them to have any contact whatsoever with the older person. However, they never found out why the family members felt that way. At times, the older person did not want their family members to know that they had access to a case manager. They believed this to be a reflection of the older person's desire to remain independent of their family members.

"You want to be independent. You don't always want your children to be involved in everything. The case manager can help me and that feels good." (Case manager 2)

Another familiar task was to strengthen the older person, which took place mainly through direct contact. This contact was usually at the older person's home during a home visit. They sometimes had to drive long distances and they organized their home visits according to location to save time. They felt they could form a more accurate opinion about the older person in their home environment and that the older persons felt comfortable in their own homes. It was also the easiest way for the older persons to communicate as they could have hearing problems that made it difficult to speak on the telephone. During the home visit they showed an interest in the older person's daily life. They also functioned as conversational support, listening and acknowledging the older person. They were 'extra eyes and ears' for the older person during care planning sessions and doctor's appointments. During these sessions they listened actively and later reminded the older persons about what had been decided. They felt the older persons trusted them and they stated that without that trust they could not perform their tasks. This trust was not gained automatically. It was built up over time as the relationship between them developed. As the case managers were able to visit the older persons in their own homes, they felt they could obtain a more genuine picture of the older person.

"If they want to share their innermost thoughts, if something has affected them or if they are sad about something, I can interpret their body language and the nuances and see how the way I express myself affects them. I feel that a face-to-face meeting is definitely the most genuine." (Case manager 7)

##### Striving to improve the health system through a new role

The participants spoke about their struggles and achievements when striving to improve the health system. They stated that they had a unique opportunity to improve parts of the municipal health system and this was unlike anything they had ever experienced before in their professions. This opportunity helped them in their endeavour to bring about improvement but also presented new challenges and they felt frustrated when they were not listened to or when the organizations did not do what had been promised. They stated that their pre-understanding of the health system was necessary, as it was vital to have experienced shortcomings in the health system in order to know what needed to be improved. However, they also felt that since they had this previous experience it made them doubt whether it was even worth the time to try to bring about change in certain areas. This was because their pre-understanding told them that some areas in the health system were almost impossible to change. Despite this pre-understanding, originating from their professional experience, they felt obligated to present these improvement areas since they were representing the older person's wishes. Their endeavour to bring about improvements took place mainly in working groups made up of representatives from the organizations involved, which were formed specifically for the CM intervention. Their endeavour was also an ongoing part of their everyday work, as it brought them into contact with health professionals. This new role presented them with a unique opportunity to improve but also presented new challenges that they needed to adjust to. When trying to adapt to these diverse conditions they needed to challenge their current professional identity.

Their everyday work included constantly gathering information about areas for improvement that could later be presented in the working groups. This information was acquired from the older persons, their family members and the health professionals. Information was acquired by being an active listener and observer during their personal contact with the older persons.

"You observe and you decide that things shouldn't be the way they are. Then you can ask questions and acquire more knowledge." (Group interview)

To make improvements, the case managers regularly went through questionnaires with the older persons during their home visits or by telephone. These questionnaires were analysed to identify areas of improvement for this specific group of older persons with multi-morbidity. The use of questionnaires was also seen as a means of mapping the older person's life situation and assessing the possible needs of older persons with multi-morbidity. They felt awkward when they asked some of the questions in the questionnaire, as the questions were not adapted to older persons and they felt they were intruding. Sometimes, all they did during the home visit was to help the older person complete the questionnaire. They also felt that the questionnaire served another purpose. By going through the questionnaire they also gained access to the older person's home and life situation.

"They help me in my role as case manager by responding to the questions. I get to know their experiences, their problems and what they feel is not working in their world. Using the questionnaire, I find out this information. It's like having an entry key." (Case manager 3)

The case managers described the working groups in the different municipalities as a unique opportunity for them to present areas for improvement to representatives from the organizations involved. These were mainly improvements on the organizational level and not the individual level. When the issue was on an individual level, it was directed specifically to the individual members of the healthcare staff. In some of the improvement areas they presented, they felt it was almost impossible to bring about any changes. However, they felt obligated to present these improvement areas as it was the older person's wishes. As they presented areas for improvement they needed to adapt to the current situation. As a result, the issues were not presented as shortcomings but simply as areas that could be improved. The case managers emphasized the importance of simply presenting the facts and not assigning blame.

"You can achieve an objective in different ways. I don't need to be rude and point out that something's wrong. You can simply say that something could be different and that it could be improved." (Case manager 7)

The case managers gathered information about organizational changes in the different municipalities from the working groups. This information was later used to update the older persons about ongoing organizational changes. The case managers stated that the working groups were a way of establishing a link between the organizations and the older persons. This link offered an opportunity to improve continuity of care for the older persons, as the representatives had different mandates that could have a direct influence on various parts of the health system in the municipality. They all believed that most of the professionals in the different organizations did the best they could. However, somewhere in this chain of organizations there was a breakdown and it was the older persons who suffered from this breakdown.

"Everyone does their part and they do it very well, but somewhere along the line it fails and I think we, the case managers, can help the individual. The chain between the municipalities and the county council needs to be complete and the working groups are an excellent way of achieving this." (Case manager 2)

As part of the improvement process, they collaborated with the different representatives in the working groups. However, this collaboration did not always work sufficiently. Sometimes they felt dejected if the representatives did not listen to them or if the attendance rate in the working groups was low. They felt frustrated when they realized the improvement did not reach those who needed it. But they also felt that the working groups were an excellent medium for improvement and things started to progress once they had presented the areas that needed to be addressed.

"We realized it was not working and we highlighted the issue, which set different processes in motion. They began to look at the issue more specifically, worked out a plan of action and began to act. It wasn't just talk, it involved a lot of work. We realized that this was the forum for presenting it." (Group interview)

When the case managers told professionals in the health system of their intention to improve certain areas they sometimes encountered suspicion regarding their role. This was experienced mainly at the start of the intervention and they felt that they were seen as a threat. They believed this stemmed from the notion that other health professionals thought they would take over their responsibilities or that they were attempting to blame them for failings within their organizations. They were questioned frequently about the role of a case manager and they even questioned themselves. They often had to explain their role and this was something completely new to them. In the past, they were used to other health professionals knowing their exact role. As they tried to make sense of their role as case manager, this challenged their current professional identity.

"My role was so obvious before that I didn't have to explain myself in different situations. You introduced yourself as a head nurse or manager and it was easy. Suddenly I have to explain who I am and my role. This has been quite difficult but I've been practising." (Group interview)

As time passed within the project they gradually felt that their role was no longer being called into question to the same extent and there was greater acceptance by the health professionals. This acceptance was described as being part of a process where other health professionals were required to adapt to a new profession that was intervening in an already well-established health system.

"When you come in and do something new, you are scrutinized by the other professionals. It's part of the process. What are you doing here? Are you taking our jobs?" (Group interview)

##### Trust is vital to advocacy

The case managers expressed mixed feelings about what it was like to represent older persons, i.e. to be their advocate. They sometimes had to trust what the older person told them and disregard their own professional values and pre-understanding of the health system. They were standing outside the health system whilst acting as an advocate for the older person. This was experienced as a new role for them. When they were given the opportunity to act as advocate they felt privileged that they had the trust of the older person. They stated that it felt natural to be an advocate and they could not imagine being anything else. However, there was also uncertainty in some situations, where they felt they could not rely on the information that was given to them by the older person, as they felt the information might not be the truth. They acknowledged the need to disregard their personal values when speaking as a representative and simply voice the opinions of the older person.

"I'm supposed to focus on what the older person wants and what the most important thing is for him. It's not my values that matter but the values of the older person. It's his claims you need to put forward." (Case manager 2)

"I see this as an enormous demonstration of trust. The degree of trust by the person is such that they have transferred something from their life to me. It is a… privilege to be in this position." (Case manager 8)

They felt that older persons trusted them to speak on their behalf since they did not belong to a profession with organizational affiliation but were outside the health system. To put forward a case they needed signed consent from the older person in order to act as their 'voice'. They acknowledged that many older persons had lost confidence in the health system and the independent position of the case managers was positive in their interaction with older persons. As a result, they often omitted to tell the older persons about their current professions. They acknowledged the need to be there purely as a case manager and not as a representative of any healthcare profession. This challenged their current professional identity. However, when representing an older person it was sometimes difficult to attach sufficient power to their words as case managers are not always recognized as a profession within the health system.

"For the older person and their family it is an advantage. I'm not an official. I'm forced to adopt a certain position. I'm neutral. Neutral through and through. And I feel they have the courage to tell me when something is not working." (Case manager 3)

When entrusted by the older person to speak on their behalf, the case managers occasionally experienced how they tried to intercede in situations that had already been resolved. The older person had previously had services performed or decisions had already been made by the organizations responsible. As the case managers had no access to health service documentation systems, all their information was given to them by the older person. This was a new situation for them since they were accustomed to having access to different documentation systems, enabling them to check if the information provided was accurate. Sometimes this situation led to misunderstandings and at times they felt foolish when they realized they were arguing for something that had already been dealt with by another health professional.

"You might perhaps phone somewhere because the older person had thought of something but then it turns out to be wrong as they might have already received the necessary help or the task in question might have been performed. Their minds are not clear and they don't really remember what was decided." (Case manager 6)

In some cases they were concerned as they had the impression that the health professionals were annoyed by the fact that they were interceding for the older person and at times it was difficult to believe the stories told by the older person. In those uncertain instances they felt that having in-depth knowledge of the older person's situation and their perceived needs made it much easier to represent them. They gathered this information from different sources and perspectives, i.e. the older person, their family members and the health professionals.

"She lives in special accommodation, where everything and everyone are wrong. She told me things that would amaze you. She said how awful everyone was and you take in everything. I listen of course but then I need to obtain an account of the situation from someone else to compare and see if it really is that horrible and that no one did anything." (Case manager 2)

## Discussion

The findings from this study illustrate that a case manager's everyday work within a CM intervention involves a challenge to their current professional identity. This challenge arises as they try to make sense of their new role as case manager in relation to their current professional identity. The concept of professional identity [38-41], is not a constant element but is more personal and complex and is shaped by contextual factors. The process of shaping an identity is affected by how the person's 'social self' is formed in new ways although values, beliefs and social and linguistic forms affect the process [41]. As the participants performed familiar work but in an unfamiliar role as case managers, they utilized their pre-understanding from their current professions as well as the knowledge residing within the case manager team. The new role as case manager exposed them to different conditions that govern how they should or should not perform the tasks. Adherence to these diverse conditions, both as an individual and as a team member, challenged their current professional identity. Clark, Hyde and Drennan [41] state that people acquire different values and perceptions from other people within the same context. This context produces a sense of what you should and should not do, i.e. what is socially appropriate [41]. The participants acquired different values and perspectives during their everyday work as case managers. They had regular discussions within the case manager team regarding the focus of their assignment and its nature. Through this interaction they were required to challenge their own values and beliefs, which in turn also challenged their current professional identity.

When the case managers adjusted to familiar work in an unfamiliar role they performed different tasks, such as navigating through the health system, planning and evaluating, strengthening the older persons and providing their families with guidance. A previous CM intervention conducted by Yau et al. [22] involved a nurse-led, telephone-based management service for frail older persons. A comparison with the results from our study revealed similarities and differences. The authors describe the importance of a trusting relationship as a means of persuading the older person to comply with a management plan [22]. Compared with our results, this trusting relationship was reported to be of greater significance, as it is this trust that helped them to guide, plan and evaluate tasks and to strengthen the older persons. They were trusted by the older persons to ensure their needs were met. This contrast could be due the fact that the case managers in our study had regular personal interaction in the form of home visits, giving them an opportunity to further their trusting relationship. Yau et al. [22] reported that the case managers sometimes experienced stress due to the unclear boundaries of professional accountability between themselves and the geriatricians. Comparing our results we acknowledge similarities, as the case managers expressed how they felt frustrated not knowing what they should or should not do in certain situations. This was due to the unclear boundaries of their professional responsibilities.

When striving to improve the health system, the participants needed to collaborate with various health professionals. Previous research [3,16,21,23] emphasizes the importance of efficient collaboration between the case manager and other health professionals in the health system. In the present study, the case managers stated that at the start of the CM intervention they often felt doubted regarding their role. They were also seen as a threat by other health professionals. As time passed, they acquired greater acceptance. This progress bears similarities to a study conducted by de Stampa et al. [21]. Initially, collaboration between the case managers and the primary care physicians (PCPs) was seen more as a hierarchical relationship. The PCPs only began to collaborate fully when they saw how the case managers could improve the quality of care. During the initial stages of their collaboration it was difficult to legitimize the role of a case manager in the eyes of the PCPs. As time passed, however, more trust was built into the relationship, which enhanced the collaboration [21].

This CM intervention (See Figure 1) aimed to improve continuity of care for this group of older persons. Hence, they were responsible for quality improvement [42] within the health system. The participants all acknowledged the importance of the working groups as a viable medium to improve continuity of care. This opinion is also consistent with other literature sources [43-47], which emphasize collaboration as an important factor in achieving qualitative improvement within the health system.

### Implications for clinical practice

This ethnographic study may help to fill knowledge gaps regarding what actually takes place in practice during a CM intervention, viewed from the case managers' perspective. There have been few studies that explore different types of CM models from the case managers' perspective [21-25]. However, this CM intervention differs in intervention design (See Figure 1) and also intervenes in a health system that was not previously accustomed to CM services. This knowledge could help policymakers in their understanding of CM and be helpful when designing a CM intervention within the health system. The case managers in this CM intervention had several opportunities to directly influence parts of the organizational structure within the health system (See Figure 1). We can see the benefits of giving case managers the opportunity to make organizational improvements as they possess a unique perspective of both the health system and the older person. This could make a contribution to existing ways of bringing about organizational improvement and thus improve the care of older persons with multi-morbidity. We also acknowledge the challenges of intervening in an already well-established health system as is the case in the Swedish context. Policymakers need to give greater consideration to supporting the work of case managers, especially at the beginning of their introduction into a health system. Finally, presenting a clear description of a case manager's professional responsibility to the health professionals in the organizations could facilitate the process of introducing case managers into a health system that is not accustomed to CM.

### Methodological considerations

We aimed to maximize data variation by attempting to include both active case managers as well as those who had previously been working as case managers within the CM intervention. One of the former case managers did not respond to the invitation, leaving one individual perspective missing from the data. We also acknowledge the limitation that this study was performed in the context of the Swedish health system, which constricts the generalizability of the findings. All data were collected by the first author who, as a registered nurse, had a pre-understanding of the context of the current health system. This pre-understanding influenced the observations as well as the research process, which can be seen as both a methodological strength and limitation. This inside perspective of the health system could have advanced data collection and analysis but it could also have clouded the observations. To address these methodological concerns, the observer (MG) regularly asked the case managers how they experienced various situations. This was done during the observation periods to explore how the case managers experienced the observed situation with regard to the observer. In the case of inconsistencies in what had been observed, the observer asked further clarifying questions to gain a deeper understanding. In addition, the first author regularly wrote field notes comprising critical reflections on his fieldwork. These field notes were frequently discussed with other authors (JK, GH, and DB) as a way of reflecting critically on the findings.

To minimize the limitations of this study, we performed triangulation [48,49]. This was done as both observations and interviews were used to gain a better understanding of the research question. Triangulation in ethnographic research [49] can help to reveal different perspectives on specific issues concerning knowledge and practice related to a specific issue. This triangulation can promote the quality of the research in ethnography [49]. Triangulation [48] was also performed as the authors (MG, JK, GH and DB) were all active during the different analytical processes, bringing their own perspectives to the data and reflecting on the findings. Furthermore, to minimize the limitations we sought to describe carefully the context, data collection and analytical process.

## Conclusions

This ethnographic study highlights case managers' experiences of their everyday work, thus providing a rich and detailed description. The findings show how their everyday work involves a challenge to their current professional identity, as they try to make sense of their role of case manager. These findings could help to understand and promote the development of CM models aimed at a population of older persons with complex health needs.

## Abbreviations

CM: Case management.

## Competing interests

The authors declare that they have no competing interests.

## Authors' contributions

MG performed the observations and conducted the individual interviews. MG and DB conducted the group interview and DB acted as moderator. MG made the analysis together with JK, GH and DB and all the authors assisted with the study design. DB contributed strongly to the study design and analysis and acted as mentor to MG, tutoring in the methodology of ethnography. JK contributed significantly to the analysis of the study. AW contributed to the study design and reviewed the manuscript. All authors read and approved the final manuscript.

## Authors' information

MG is a PhD student, registered nurse and works as a lecturer. JK is a registered nurse, holds a PhD in nursing and works as a senior lecturer. GH is Associate Professor of Care Science, works as a senior lecturer and was involved in the initial design of the Blekinge CM intervention. AW is a registered nurse and Professor of Care Science. DB is a registered nurse, ethnographer, holds a PhD in nursing and works as a senior lecturer.

## Pre-publication history

The pre-publication history for this paper can be accessed here:

http://www.biomedcentral.com/1472-6963/13/496/prepub

## References

[B1] You EC Dunt D Doyle C Hsueh A Effects of case management in community aged care on client and carer outcomes: a systematic review of randomized trials and comparative observational studies BMC Health Serv Res 2012 13 395 10.1186/1472-6963-12-395 23151143 PMC3508812

[B2] Long MJ Case management model or case manager type? That is the question Health Care Manag (Frederick) 2002 13 53 65 12083179 10.1097/00126450-200206000-00009

[B3] Oeseburg B Wynia K Middel B Reijneveld SA Effects of case management for frail older people or those with chronic illness: a systematic review Nurs Res 2009 13 201 210 10.1097/NNR.0b013e3181a30941 19448524

[B4] Gravelle H Dusheiko M Sheaff R Sargent P Boaden R Pickard S Parker S Roland M Impact of case management (Evercare) on frail elderly patients: controlled before and after analysis of quantitative outcome data BMJ 2007 13 31 10.1136/bmj.39020.413310.55 17107984 PMC1764106

[B5] Naylor MD Brooten D Campbell R Jacobsen BS Mezey MD Pauly MV Schwartz JS Comprehensive discharge planning and home follow-up of hospitalized elders: a randomized clinical trial JAMA 1999 13 613 620 10.1001/jama.281.7.613 10029122

[B6] Markle-Reid M Weir R Browne G Roberts J Gafni A Henderson S Health promotion for frail older home care clients J Adv Nurs 2006 13 381 395 10.1111/j.1365-2648.2006.03817.x 16629922

[B7] Swedish National Institute of Public Health Healthy Ageing – A Challenge for Europe 2007 Stockholm: Swedish National Institute of Public Health

[B8] Boyd CM Darer J Boult C Fried LP Boult L Wu AW Clinical practice guidelines and quality of care for older patients with multiple comorbid diseases: implications for pay for performance JAMA 2005 13 716 724 10.1001/jama.294.6.716 16091574

[B9] Marengoni A Winblad B Karp A Fratiglioni L Prevalence of chronic diseases and multimorbidity among the elderly population in Sweden Am J Public Health 2008 13 1198 1200 10.2105/AJPH.2007.121137 18511722 PMC2424077

[B10] Fortin M Soubhi H Hudon C Bayliss EA van den Akker M Multimorbidity’s many challenges BMJ 2007 13 1016 1017 10.1136/bmj.39201.463819.2C 17510108 PMC1871747

[B11] Summer Meranius M Era delar är min helhet – en studie om att vara äldre och multisjuk PhD thesis 2010 Växjö, Sweden: Linnaeus University

[B12] Bayliss EA Edwards AE Steiner JF Main DS Processes of care desired by elderly patients with multimorbidities Fam Pract 2008 13 287 293 10.1093/fampra/cmn040 18628243 PMC2504745

[B13] Westra BL Cullen L Brody D Jump P Geanon L Milad E Development of the home care client satisfaction instrument Public Health Nurs 1995 13 393 399 10.1111/j.1525-1446.1995.tb00168.x 8545307

[B14] Golden AG Tewary S Dang S Roos BA Care management’s challenges and opportunities to reduce the rapid rehospitalization of frail community-dwelling older adults Gerontologist 2010 13 451 458 10.1093/geront/gnq015 20185522

[B15] Swedish National Institute of Public Health Anhöriga som ger omsorg till närstående – omfattning och konsekvenser 2012 Stockholm: Swedish National Institute of Public Health

[B16] Eklund K Wilhelmson K Outcomes of coordinated and integrated interventions targeting frail elderly people: a systematic review of randomised controlled trials Health Soc Care Community 2009 13 447 458 10.1111/j.1365-2524.2009.00844.x 19245421

[B17] Case Management Society of America Standards of Practice for Case Management 2010 3 Little Rock, Arkansas: Case Management Society of America

[B18] Zawadski RT Eng C Case management in capitated long-term care Health Care Financ Rev 1988 Spec No:75–81 PMC4195120 10312976

[B19] Capitman JA Haskins B Bernstein J Case management approaches in coordinated community-oriented long-term care demonstrations Gerontologist 1986 13 398 404 10.1093/geront/26.4.398 3089878

[B20] Coleman EA Parry C Chalmers S Min S-J The care transitions intervention: results of a randomized controlled trial Arch Intern Med 2006 13 1822 1828 10.1001/archinte.166.17.1822 17000937

[B21] De Stampa M Vedel I Bergman H Novella J-L Lechowski L Ankri J Lapointe L Opening the black box of clinical collaboration in integrated care models for frail, elderly patients Gerontologist 2013 13 313 325 10.1093/geront/gns081 22961463

[B22] Yau DCN Leung ACT Yeoh C-S Chow NWS Global case management: Hong Kong. Care for the hospital-discharged frail elders by nurse case managers: a process evaluation of a longitudinal case management service project Lippincotts Case Manag 2005 13 203 212 16056117 10.1097/00129234-200507000-00006

[B23] Mackenzie A Lee DT Dudley-Brown S Chin TM Case management in Hong Kong: evaluation of a pilot project in community nursing J Clin Nurs 1998 13 291 292 10.1046/j.1365-2702.1998.00177.x 9661393

[B24] Black K Fauske J Exploring influences on community-based case managers’ advance care planning practices: facilitators or barriers? Home Health Care Serv Q 2007 13 41 58 10.1300/J027v26n02_03 17537710

[B25] Skillen DL Anderson MC Knight CL The created environment for physical assessment by case managers West J Nurs Res 2001 13 72 89 10.1177/01939450122044961 11216026

[B26] Hallberg IR Kristensson J Preventive home care of frail older people: a review of recent case management studies J Clin Nurs 2004 13 112 120 10.1111/j.1365-2702.2004.01054.x 15724826

[B27] Cruz EV Higginbottom G The use of focused ethnography in nursing research Nurse Res 2013 13 36 43 23520711 10.7748/nr2013.03.20.4.36.e305

[B28] Speziale HJS Streubert HJS Carpenter DR Qualitative Research in Nursing: Advancing the Humanistic Imperative 2011 Philadelphia: Lippincott Williams & Wilkins

[B29] Morse JM Critical Issues in Qualitative Research Methods 1994 Thousand Oaks, Calif: SAGE

[B30] Galanti GA How to do ethnographic research West J Med 1999 13 19 10483338 PMC1305726

[B31] Polit DF Beck CT Nursing research : generating and assessing evidence for nursing practice 2012 Philadelphia: Wolters Kluwer Health/Lippincott Williams & Wilkins

[B32] National Board of Health and Welfare End of life care: The National Board of Health and Welfares assessment of the development in county council and municipal 2006 Stockholm: National Board of Health and Welfare

[B33] Angrosino M The SAGE Qualitative research kit 3 Doing ethnographic and observational research 2007 London: SAGE

[B34] Kvale S InterViews: learning the craft of qualitative research interviewing 2009 2 Los Angeles: Sage Publications

[B35] Morse JM Field PA Qualitative Research Methods for Health Professionals 1995 Thousand Oaks, Calif: SAGE

[B36] Klein B Transkribering är en analytisk akt In Rig 1990 13 41 66

[B37] WMA Declaration of Helsinki Ethical Principles for Medical Research Involving Human Subjectshttp://www.wma.net/en/30publications/10policies/b3/index.html 19886379

[B38] Adams K Hean S Sturgis P Clark JM Investigating the factors influencing professional identity of first-year health and social care students Learn Health Soc Care 2006 13 55 68 10.1111/j.1473-6861.2006.00119.x

[B39] Hotho S Professional identity – product of structure, product of choice: linking changing professional identity and changing professions J Organ Chang Manag 2008 13 721 742 10.1108/09534810810915745

[B40] Pratt MG Rockmann KW Kaufmann JB Constructing professional identity: the role of work and identity learning cycles in the customization of identity among medical residents Acad Manage J 2006 13 235 262 10.5465/AMJ.2006.20786060

[B41] Clarke DM Hyde A Drennan J Kehm BM, Teichler U Professional identity in higher education The Academic Profession in Europe: New Tasks and New Challenges 2013 Netherlands: Springer 7 21

[B42] Batalden PB Davidoff F What is “quality improvement” and how can it transform healthcare? Qual Saf Health Care 2007 13 2 3 10.1136/qshc.2006.022046 17301192 PMC2464920

[B43] Cronenwett L Sherwood G Barnsteiner J Disch J Johnson J Mitchell P Sullivan DT Warren J Quality and Safety Education for Nurses Nurs Outlook 2007 13 122 131 10.1016/j.outlook.2007.02.006 17524799

[B44] van Bokhoven MA Kok G van der Weijden T Designing a quality improvement intervention: a systematic approach Qual Saf Health Care 2003 13 215 220 10.1136/qhc.12.3.215 12792013 PMC1743716

[B45] Thor J Wittlöv K Herrlin B Brommels M Svensson O Skår J Øvretveit J Learning helpers: how they facilitated improvement and improved facilitation–lessons from a hospital-wide quality improvement initiative Qual Manag Health Care 2004 13 60 74 10.1097/00019514-200401000-00006 14976908

[B46] Shojania KG Grimshaw JM Evidence-based quality improvement: the state of the science Health Aff 2005 13 138 150 10.1377/hlthaff.24.1.138 15647225

[B47] Lukas CV Holmes SK Cohen AB Restuccia J Cramer IE Shwartz M Charns MP Transformational change in health care systems: an organizational model Health Care Manage Rev 2007 13 309 320 10.1097/01.HMR.0000296785.29718.5d 18075440

[B48] Creswell JW Qualitative Inquiry and Research Design: Choosing Among Five Approaches 2012 Thousand Oaks: SAGE Publications

[B49] Flick U Managing Quality in Qualitative Research 2007 Thousand Oaks, Calif: SAGE

